# Trial Proteomic Qualitative and Quantitative Analysis of the Protein Matrix of Submandibular Sialoliths

**DOI:** 10.3390/molecules26216725

**Published:** 2021-11-06

**Authors:** Paulina Czaplewska, Aleksandra E. Bogucka, Natalia Musiał, Dmitry Tretiakow, Andrzej Skorek, Dominik Stodulski

**Affiliations:** 1 Intercollegiate Faculty of Biotechnology, University of Gdansk, 80-307 Gdansk, Poland; lewand.ola@gmail.com (A.E.B.); n.musial.773@studms.ug.edu.pl (N.M.); 2 Department of Otolaryngology, Medical University of Gdansk, 80-214 Gdansk, Poland; d.tret@gumed.edu.pl (D.T.); askorek@gumed.edu.pl (A.S.); dstodulski@gumed.edu.pl (D.S.)

**Keywords:** sialolith, mass spectrometry, proteomics, FASP, SWATH

## Abstract

Our studies aimed to explore the protein components of the matrix of human submandibular gland sialoliths. A qualitative analysis was carried out based on the filter aided sample preparation (FASP) methodology. In the protein extraction process, we evaluated the applicability of the standard demineralization step and the use of a lysis buffer containing sodium dodecyl sulfate (SDS) and dithiothreitol (DTT). The analysis of fragmentation spectra based on the human database allowed for the identification of 254 human proteins present in the deposits. In addition, the use of multi-round search in the PEAKS Studio program against the bacterial base allowed for the identification of 393 proteins of bacterial origin present in the extract obtained from sialolith, which so far has not been carried out for this biological material. Furthermore, we successfully applied the SWATH methodology, allowing for a relative quantitative analysis of human proteins present in deposits. The obtained results correlate with the classification of sialoliths proposed by Tretiakow. The performed functional analysis allowed for the first time the selection of proteins, the levels of which differ between the tested samples, which may suggest the role of these proteins in the calcification process in different types of sialoliths. These are preliminary studies, and drawing specific conclusions requires research on a larger group, but it provides us the basis for the continuation of the work that has already begun.

## 1. Introduction

The human body produces various body fluids throughout its life, in which, except for the supersaturated state, the rich composition of inorganic salts and proteins/peptides remains stable. This condition is maintained thanks to the presence of stabilizing agents. For example, in human milk, casein is such a protein, which stabilizes the high concentration of calcium phosphate (CaP) [1]. In human saliva, this function is performed by statherin (STATH) and acidic proline-rich peptides (aPRPs) [2]. However, in certain situations, despite the presence of stabilizers, homeostasis may be disturbed, and in the salivary ducts and/or in salivary glands, deposits called salivary stones or sialoliths may form. A pathological condition characterized by the formation of a sialolith in the salivary gland is called sialolithiasis. In such a pathological situation of saliva retention, dilatation of the salivary ducts and often an active inflammatory process can be observed. It is a rare, poorly described, and less known situation than popular urolithiasis or gallstones, and it affects 1 per 15,000 up to 1 per 30,000 of the population [3]. Sialolithiasis is encountered in the clinical practice of otolaryngologists, stomatologists, and maxillofacial surgeries. This pathologic condition is more common in the submandibular (4–6:1) than the parotid and sublingual salivary glands [4,5,6]. The most common symptoms of this condition include sudden pain in the salivary glands’ region, escalating during a meal, swelling, and a fever. A higher mucus concentration in the submandibular gland saliva and the intricate long winding course of the salivary gland duct can predispose to sialolithiasis. Other predisposing factors include tobacco smoking, caries, reduced fluid intake, and medications that diminish salivary output [7,8,9]. Among the potential causes are injury and the salivary glands’ inflammation, precipitation of salts, and dental and endocrine disease. Moreover, the other origins of sialolithiasis are microliths around which calcium crystals can deposit, alkalinity of saliva causing precipitation of calcium phosphate, oral bacteria, and migration of food debris into the salivary duct [10,11]. It suggests that the whole process is rather multifactorial than being caused by one particular factor or event.

So far, research on this disease has been focused on imaging techniques allowing surgeons to determine the location of the sialolith before surgery and the treatment methods, during which the salivary stone is removed and the patient returned to regular activity. However, imaging examinations do not allow finding out the exact aetiology of the formation of deposits in the salivary ducts. Therefore, there are no methods of predicting the disease, and there are no prophylactic or non-surgical treatments for patients. Progress is essential because sialolithiasis tends to recur, and surgical treatment (removing the stone by endoscopy or transoral or transcervical surgery) is not a final treatment. In some cases, to heal a patient, the salivary gland must be resected radically. Thus, it is crucial to understand the mechanism of biocalcification and the role of the peptides and proteins, mainly belonging to the calcium stabilizers family, in the biomineralization process occurring in the sialolithiasis.

The structure of sialoliths is variable. The typical salivary calculi contain a round core, surrounded by thin inorganic and organic layers composed of mucin, proteins, carbohydrates, lipids, bacterial cells, and mineral constituents, especially apatites [12,13,14,15]. The inorganic part of sialoliths has been studied quite precisely, while the organic portion and the protein/peptide content are currently at the early research stage. The latest classification of sialoliths—proposed by Tretiakow et al. and based on the spectroscopic studies of the core structure of the calculi—divides salivary stones into three types: calcified (CAL), organic/lipid (LIP), and mixed (MIX) ones [16]. The structure analysis of the core and inner layers showed a similarity in the central part for CAL and MIX type, while CAL and LIP have their separate path of origin and development. These results may suggest that disturbances in regulating the balance of calcium or lipids may play a role in the first stage of such deposit formations. The changes in the levels or structure of proteins/peptides secreted by the salivary glands and responsible for the ion binding, transport, and deposition can be a trigger factor for the biocalcification process. Therefore, it is important to determine whether disturbances in the function or levels of proteins regulating calcium and lipid metabolism may be a factor triggering the process of calcium deposition in the case of sialoliths of the CAL and MIX type, or lipids in the case of the LIP type.

Scientific work aimed at the analysis of proteins deposited in sialoliths is not carried out intensively. Most of the work is focused on urinary stones formed in the bladder or urinary duct, and the number of publications per year is about fifteen. In recent years, only one work in the literature was dedicated to proteomics and proteins extraction from sialoliths [17] and one paper was dedicated to microbiota analysis in sialolithiasis [17]. In the first paper, Busso et al. focused on studying 29 sialoliths using mass spectrometry and global statistical analysis of the results. They compared several protein extraction protocols in their work, and the obtained protein fractions were subjected to the standard trypsin in solution digestion procedure and LC-MS/MS analysis. To the best of our knowledge, it is the first proteomic protocol for sialolith analysis described in the literature.

Inspired by the results of the work by Busso et al., we decided to check the application of the FASP methodology and the lysis buffer used in this procedure, combined with demineralization for extraction and further proteomic analysis using mass spectrometry. In the qualitative analysis, we applied a two-step approach to analysing the results—processing the results with the human protein database, followed by analysis with a bacterial database of previously unassigned results. We also carried out the first approach to sialolith protein quantification using the SWATH-MS method. We selected the first potential marker proteins characteristic of the stone formation process in salivary glands.

## 2. Results

### 2.1. Classification and Qualitative Analysis of Sialoliths Protein Matrix

**Figure 1:**
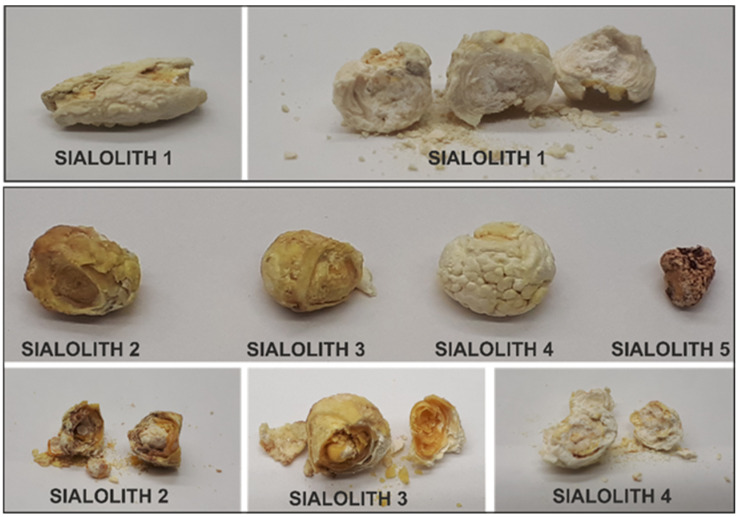
Pictures of sialoliths used for qualitative and quantitative research.

The pilot studies were carried out on a group of five sialoliths, which are shown in Figure 1. The stone marked Sialolith 1 in Figure 1 was selected for testing the protein extraction protocols, and the remaining four samples, marked Sialolith 2–5, were used for the quantitative pilot studies.

**Figure 2:**
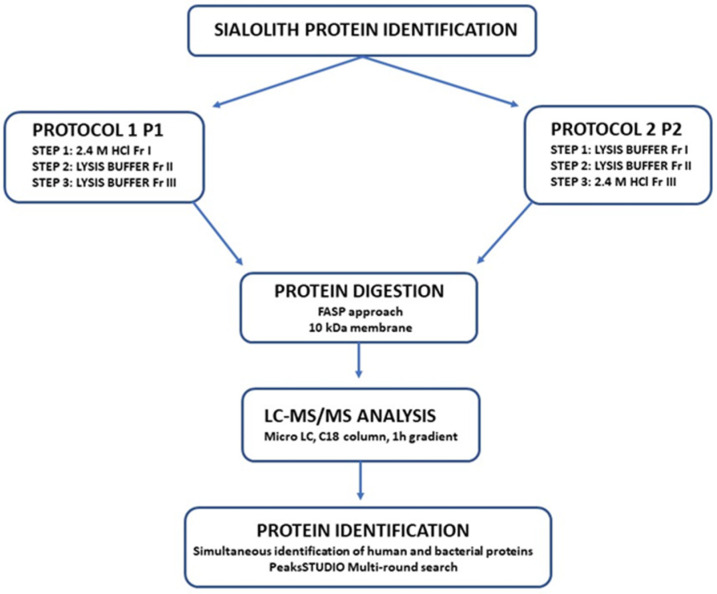
The diagram represents the methods used to identify sialolith constituent proteins.

To extract proteins from sialoliths, we used two protocols, which are summarized in Figure 2. For testing the methods, we chose one of the stones marked as S1 and, after pre-crushing it into smaller pieces, it was pulverized in a ceramic mortar in liquid nitrogen. It allowed us to obtain a fine powder, from which two samples of 200 mg of material each were prepared. The first batch was dedicated to protocol 1, which started with the frequently used 2.4 M hydrochloric acid demineralization. For the second lane, powdered sialolith was treated directly with a lysis buffer conventionally used in the filter-assisted sample preparation (FASP) procedure. For example, in protocol 1 (P1), the hydrochloric acid-containing fraction (P1 Fr I) was obtained after decanting the sialolith suspension and water fraction after washing demineralized powder from acid (P1 Fr II) was transferred separately to 3 kDa Microcons. Furthermore, it was washed with lysing buffer in five cycles and then heated for 10 min at 95 degrees so that all solutions were treated the same (reduced with DTT and well dissolved) before digestion. The powder remaining after demineralization and washing was treated twice with the lysis buffer. Each time after adding the solution, the whole sample was heated for 10 min at 95 degrees Celsius and decanted after cooling. In this way, we obtained the following fractions marked as P1 Fr III. In an extraction attempt starting with the lysis buffer, a sample of the comminuted sialolith was treated twice with the lysis buffer (P2 Fr I and P2 Fr II). Finally, we additionally demineralized with 2.4 M hydrochloric acid to obtain the P2 Fr III fraction. For this fraction, we dialyzed the lysis buffer in the same way as for the P1 Fr I fraction.

Protein digestion for each of the received fractions separately was performed according to the standards of the FASP procedure. After heating the proteins in the lysis buffer and cooling the solution, protein concentration was measured, and in each case, 100 µg of proteins was taken from the tested fraction and placed on a 10 kDa membrane. The trypsin digestion was performed overnight in a 37 °C constant temperature incubator. After digestion, 10 µg of peptides from each fraction was subjected to final StageTips purification with C18 phase. After LC-MS/MS analysis according to the parameters described in the Materials and Methods chapter, the obtained data were analysed in the PEAKS Studio program. First, the data were analysed against the *Homo sapiens* database, and then in Multi-Round Search were analysed against the entire bacterial database. In this way, identifications were performed for each of the fractions obtained in the protein extraction process. Detailed results of the analysis for sample S1 are presented in Supplementary Table S1 in the manuscript supplement.

**Figure 3:**
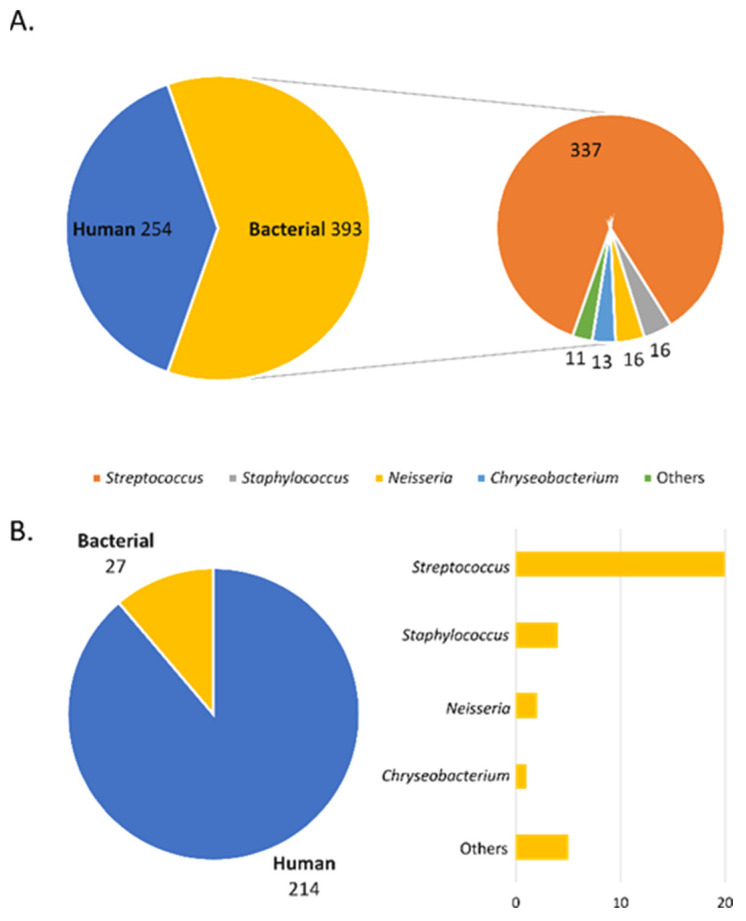
Pie charts with all identified proteins identified for human and bacterial databases: (**A**) total human and bacterial proteins; (**B**) proteins identified in groups specifying the type of bacteria in the bar chart.

The conducted experiments allowed the identification of about 600 proteins at 1% FDR, of which 245 were human proteins, and the remaining 393 were bacterial proteins (Figure 3A). Analysing the number of proteins for individual fractions shows that apart from human proteins, bacterial proteins were identified in each fraction. However, there is a clear difference in the amount of identified bacterial proteins in individual fractions. Most of the bacterial proteins are present in fractions II and III of both protocols where the lysis buffer was used. In both cases, the second treatment of the sialolith suspension with buffer resulted in a significant increase in the amount of identified bacterial proteins. Analysing the type of bacteria from which the proteins were identified, they were mainly *Staphylococcus*, *Streptococcus*, *Neisseria*, and *Chryseobacterium* (Figure 3B).

### 2.2. Trial of Quantitative Analysis of Proteins of Sialoliths S2–S5

**Figure 4:**
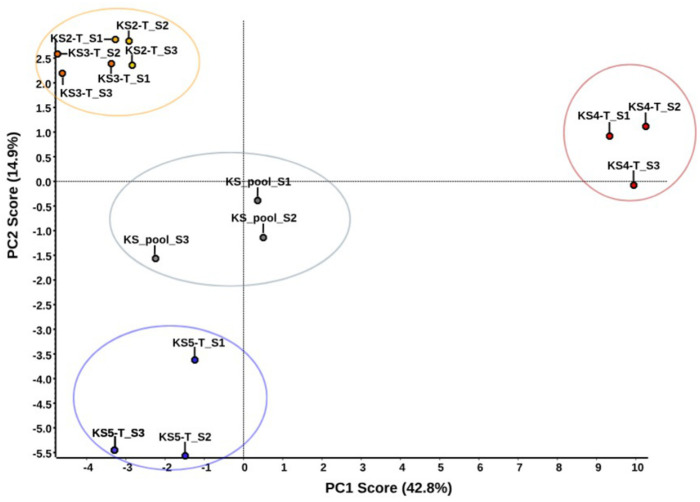
PCA analysis of four sialoliths used in quantitative analysis.

The analysis of sialoliths S2–S5 was performed according to protocol 1. The amount of starting material (powdered sialolith) was determined by S5, which was the smallest stone submitted for proteomic studies (Figure 1). Weights of 90 mg of each of them after extraction were digested according to the MED-FASP protocol. Qualitative analysis was performed in the PEAKS Studio software for each of the analysed sialoliths S2–S5 (Supplementary Table S2). It was possible to identify 173 proteins for S2, 160 proteins for S3, 210 proteins for S4, and 155 proteins for S5. No non-human proteins were found. The first step in the quantitative analysis in the SWATH approach was the preparation of a peptide library based on the results of the qualitative analysis. It was created in the joint database search for S2 to S5 samples, at 1% FDR. The library used for the analysis consisted of 150 human proteins. According to the conducted statistical analysis assumptions, all proteins with a concentration change described by *p*-value < 0.05 were considered to be present at statistically significant different concentrations. Principal component analysis (PCA) was carried out for the four sialoliths tested and for the pooled sample used to create the library. The result is presented in Figure 4, which shows a clear division into groups of the examined samples. Sialoliths S2 (KS2-T) and S3 (KS3-T) are very similar to each other and form one cluster, S5 (KS5-T) separates from the rest, and a big difference is visible in the case of the S4 (KS4-T) sialolith.

Each sample of a tested sialolith was individually compared with the combined four samples (pool). In all investigated statistical comparisons, we detected 34 differential proteins (Table 1).

## 3. Discussion

Identification of proteins from human biological material is in most cases associated with work with tissue obtained during medical examinations or surgery; from body fluid, such as blood/plasma/cerebrospinal or fluid/tears/breast milk; or with cell lines. However, the research also includes more demanding material, such as amyloid deposits or even fragments of bone tissue, and forms such as stones. Here, the extraction of proteins requires more extreme reagents, most of which are not friendly to the mass spectrometry used for spectra recording. In our work, we examined salivary stones, which are not a frequent source of proteins for analysis. Due to their mineral composition, the first step in the few proteomics studies devoted to them has been the use of concentrated hydrochloric acid and demineralization. The process is effective. However, it is not indifferent to both proteins and laboratory devices. Therefore, the possibility of skipping this step is always welcome in all protein extraction protocols. In our case, we also decided to check the possibility of skipping this stage by replacing it with extraction with a lysis buffer based on its composition of sodium dodecyl sulfate and dithiothreitol. It is the solution recommended in the FASP procedure, which is currently commonly used by many laboratories to digest protein mixtures. Thus, we compared two protocols, one with classical demineralization and then a two-stage protein extraction with lysis buffer with the protocol where we first used two-stage extraction and finished with the final demineralization of the residue. Each fraction was processed following the FASP procedure, and the final framing spectra were used for protein identification. In the last step, we also decided to check whether the sequential search of databases would identify human and bacterial proteins from one set of data. We chose the PEAKS Studio program for the analysis, where we entered the *Homo sapiens* database as the first and the bacterial base as the subsequent database for identification of unassigned spectra. The results show that after crushing the salivary stone, the demineralization process was not crucial for protein extraction. The use of two successive extractions with the lysis buffer allowed the extraction of a significant amount of protein. This process, of course, can still be refined, and when used in the SDS buffer at a concentration of 1%, it gives room for manoeuvre. Its concentration can be increased to 4%. The identification of bacterial proteins indicated that these were also good conditions for obtaining bacterial proteins. They would mostly be destroyed in the cellular structure when heated in the lysis buffer, releasing their contents into the solution. Thus, it was a good approach if we had the right amount of stone material and the bacterial cells were present in it, which may have resulted from their participation in the formation of deposits. Due to the amount of material, we could not check all the modifications that came to our minds on one selected stone. Here, the work continued in our laboratory.

**Figure 5:**
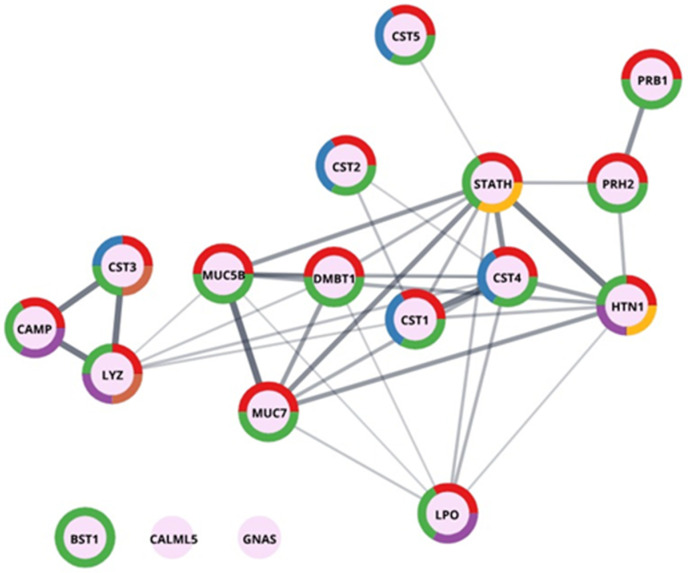
Graphic presentation of GO enrichment in the category—salivary proteins for sialolith1. The colours depicted in the circles correspond accordingly to Uniprot keywords enrichment: red—secreted, blue—thiol protease inhibitor, green—signal, purple—antimicrobial, brown—amyloid, yellow—biomineralization.

**Figure 6:**
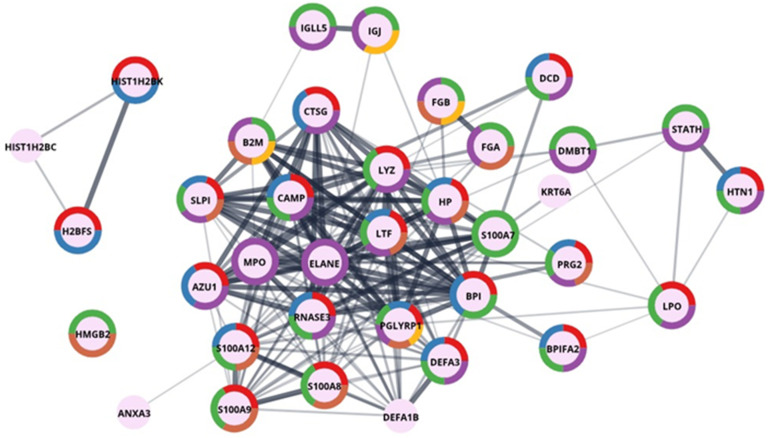
Graphic presentation of GO enrichment in the category—defence response to bacterium-associated proteins for sialolith1. The colours depicted in the circles correspond accordingly to protein function enrichment according to Uniprot keywords enrichment: red—antimicrobial, blue—antibiotic, green—secreted, purple—signal, brown—immunity, yellow—pyrrolidone carboxylic acid.

*Human proteins in salivary stones.* In qualitative studies, we identified a total of 245 human proteins (214 in groups) for sialolith S1. Figure 5 shows the results of performed GO enrichment in the main category—salivary proteins, and Figure 6 in the category—defence response to bacterium-associated proteins. The presented proteins were colour-coded by function into categories such as secreted proteins, thiol protease inhibitors, signalling proteins, antimicrobial proteins, and amyloid and proteins involved in biomineralization processes. Considering the oral cavity environment of the sialoliths formation, this was quite an expected result. Most of the proteins identified and shown in Figure 5 are multifunctional, suggesting a complex process influenced by many factors.

The first thing that catches your eye is the presence of a wide variety of proteins from the group of serine protease inhibitors—namely, cystatins (CYST1-5). They are responsible for regulating the activity of enzymes from the cathepsin group. They control the process of protein degradation in the human body and thus also in the oral cavity [18]. However, this is not the only function of cystatin proteins. They are present in all body fluids, and it is known that they may also be an organism’s response to the appearance of pathogenic microorganisms, or in truncated forms (such as cystatin S, CYST4), they may be involved in the maintenance of calcium–phosphate homeostasis [19]. In the longer term, the imbalance may be related to the remineralization processes of the tooth, the formation of the pellicle, and the prevention of dental caries [19].

On the other hand, cystatin C (CST3) is also known as an amyloidogenic protein. The L68Q mutation causes Icelandic-type amyloidosis, plaque build-up, cerebral haemorrhages, and the death of young people [20]. Non-mutated protein, with changing conditions, e.g., lowering the pH, may also oligomerise. It also willingly interacts with other proteins, e.g., the serum amyloid A (SAA) [21,22] or the amyloid b peptide [23]. Therefore, it is possible that the presence of this group of proteins, by binding partners, causes their coprecipitation in the sialolith, analogous to the cerebral deposits.

Another prominent group are proteins, which are the body’s defence against different types of external pathogens or excessive growth of pathogens present in the oral cavity. The identified antimicrobial proteins include histatin (HTN1), lactoperoxidase (LPO), lysozyme (LYZ), and cationic antimicrobial peptide (CAMP). LPO is a crucial protein for maintaining the bacterial balance of the oral cavity; it catalyses the reaction of the formation of hypothiocyanate penetrating the cells of microorganisms [24]. It is worth paying attention to the deleted in malignant brain tumour 1 protein (glycoprotein 340, salivary agglutinin, DMBT1) identified in the deposits and presented in Figure 5. Literature reports indicate the ability of this protein to bind to bacteria surfaces from the Staphylococcus group [25]. Similar properties are mentioned in the literature for lysozyme C, mucin-7, immunoglobulins, and proteins from the S100 group, such as S100-A9 [26,27]—all of them were identified as protein components of the sialolith matrix. The antibodies and proteins from the S100 group, such as the S100-A9 mentioned above and S100-A6, S100-A8, S100-A11, and S100-A12, can be found in Supplementary Table S1.

In this case, one cannot fail to mention all of the proteins involved to a greater or lesser extent in maintaining the balance of minerals and protecting salivary glands and the oral cavity against spontaneous crystallisation. In this group of proteins, statherin (STATH) is noted, which is responsible for stabilising saliva supersaturated with calcium salts by inhibiting the precipitation of calcium phosphate salts [28]. It also modulates hydroxyapatite crystal formation on the tooth surface. A second protein, histatin (HTN1), is a salivary protein considered a major precursor of the protective proteinaceous structure on tooth surfaces (enamel pellicle) [29]. In addition, histatin and statherin exhibit antibacterial and antifungal activities [30]. Another protein, calmodulin-like protein 5 (CALML5), plays a role in calcium-binding, intracellular signalling, and keratinocyte differentiation [31]. Additionally important are proline-rich peptides such as the PRB1 and PRH2 identified in salivary deposits, and they modulate calcium phosphate chemistry in the oral cavity and can modulate bacterial colonization [32,33]. The last of the proteins listed is a calcium-binding protein that functions as a proinflammatory mediator in acute and chronic inflammation.

*Bacterial proteins in sialoliths.* Comparing the identified bacteria from our list with other data in the literature, e.g., from the De Grandi publication, the authors identified bacteria from sialoliths based on bacterial genetic DNA isolated from biological material. We did not see any rare exceptions. With the help of proteomic tools, without the need to use commercial kits to isolate bacterial genetic material, obtain similar information, and simultaneously identify human proteins deposited in the stone of the bacteria identified in the sialolith, the first two belonged to the Gram-positive family Staphylococcaceae. Both of them—namely, coagulase-positive *Staphylococcus aureus* and coagulase-negative *Staphylococcus epidermidis*—are part of the normal bacterial flora of human skin and mucosal surfaces [34,35,36]. These bacteria live on human skin and mucous membranes (*S. aureus* in the nose) without causing any symptoms in healthy people. However, when they enter the blood or tissues, and the patient is immunocompromised, they find the ideal environment for uncontrolled growth. They cause severe infections (especially severe in the case of MRSA, methicillin-resistant *Staphylococcus aureus* strains). Their presence in the salivary glands, which can provide an ideal environment for uncontrolled growth, can have serious consequences. Whether overgrowth of staphylococci may be the cause of salivary stone formation is an unanswered question. The following and most numerous groups of bacteria present in sialoliths are Gram-positive and nonmotile streptococci. Based on the division into types, we identified the pyogenic type *(S. porcinus* [37] and *S. pseudoporcinus* [38]), the mitis type (*S. pneumoniae*, *S. mitis, S. oralis and S. parasanguinis* [39]) [40], the sanguinis type (*S. sanguinis* [41], *S. gordoni* [42], *S. cristatus* [43]), and the sinensis type (*S. sinensis* [44]). *S. porcinus* and *S. pseudoporcinus* are rarely identified in humans, and if anything, their presence is associated with the occupation of the rectum and vagina in women of reproductive age. This situation may also be caused by misidentification as *S. agalactitae*, also present in the vaginal–rectal specimen [45]. Only a few reports of infections caused by the presence of *S. pseudoporcinus* have appeared in the literature so far. They were a wound infection of a finger due to trauma [46], another with left lower leg cellulitis associated with stasis dermatitis [47], and two new cases: endocarditis and a second with pneumonia and empyema [48]. Specific identification and differentiation of *S. porcinus* from *S. pseudoporcinus* are complicated. Only genetic confirmation can give us certainty about which or both of them are present in salivary glands. Our results clearly show that this type of bacteria can be found in humans in the mouth and salivary glands and that it is related to infections. Comparing genetic and functional properties, this species can be classified in this respect between the typical commensal *S. mitis* and an important human pathogen *S. pneumoniae* [38]. It is found to be associated with chronic obstructive pulmonary disease [49].

Among the other identified bacteria, we can distinguish bacteria from the *Neisseria* and *Chryseobacterium* groups. For the first type, the proteins for *N. flavescens*, *N. perflava*, and *N. subflava* were identified. According to the data in the literature on the classification of the Neisseria genus based on studies of genetic material, the identified types share a large proportion of alleles and are considered variants of the same species [50]. The proteins identified for the *Chryseobacterium* genus have been assigned to different species in this group. They are uncommon human pathogens and rather associated with nosocomial infections (contaminated medical devices) [51]. Usually, these bacteria are found in the environment (water, plants) and are resistant to many factors and classified as multidrug-resistant bacteria [52]. Therefore, they are potential sources of pathological conditions, but their role in oral cavity infections, and in the process of the formation of deposits in the salivary glands, is not described and requires further research.

*Protein quantification trial.* Relative quantitative analysis based on the SWATH MS methodology was carried out as a pilot study. The key stage was the creation of a protein library, which in the next stage served the identification and quantification of spectra registered in the SWATH format, in this case with variable windows (see the Section 4) for individual sialoliths. The comparison was made between the individual trials. It was not possible to apply a control in this case; it was difficult to decide what would be the best control for sialoliths. Busso used the maxillary bone and tooth as controls for comparisons in his work [53]. However, it is difficult to imagine collecting such material from every patient with salivary duct stones. In the future, we plan to use patients’ saliva as a control and material that is easy to obtain from each patient. Therefore, we decided to base our analysis on the comparison of results between individual sialoliths. The results of the principal component analysis (PCA) (Figure 4) show four clusters for the tested samples. The samples marked as S2 and S3 (KS2 and KS3 on the Figure 4) are grouped together. S5 differs from the first cluster based on PC2 scores, and S4 differs from the rest of both PC1 and PC2, but the most important is the PC1 score. The last cluster is the mixed samples used to build the library, which are grouped in the middle of the graph as expected. Based on the classification proposed by Tretiakow [16], the sialolith S4 was classified as calcified (CAL) type. Figure 1 clearly shows the differences in colour and structure, which was also confirmed in the sample preparation process. It was almost completely dissolved under the influence of hydrochloric acid. The others can be classified as organic/lipid (LIP) type for S2 and S3 and mixed type (MIX) for S5.

**Figure 7:**
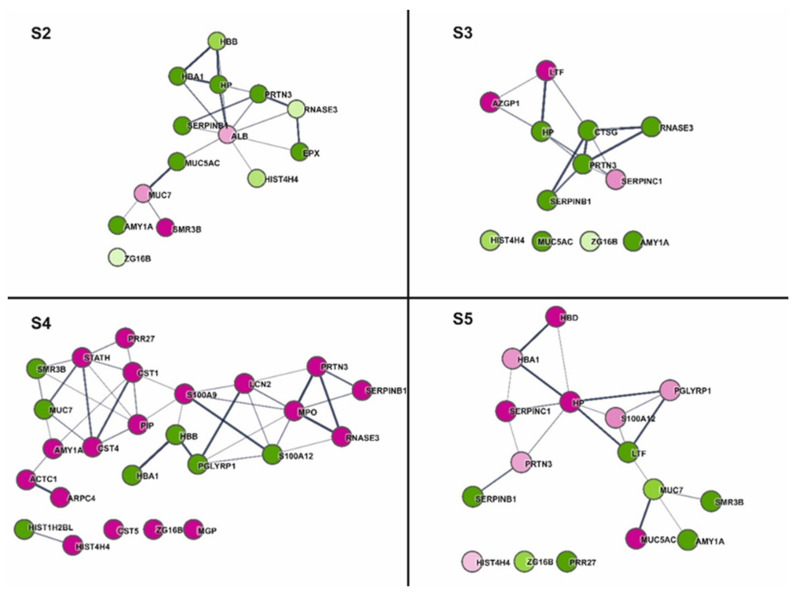
The Cytoscape visualization of STRING-generated network composed of experimentally verified protein−protein interactions among the statistically significant (*p*-value < 0.05) quantified proteins for each analysed sample (**S2**–**S5**). The gradation of the fill corresponds to the value of the concentration fold changes. Thus, the darker the fill, the more significant the difference; shades of magenta and green correspond to increased and decreased concentration in the test group, respectively.

Comparing the functional analysis results performed in our studies for the four sialoliths, we see an analogous division between sialoliths (Figure 7). S2 and S3 are mainly characterized by down-regulated proteins involved in the body’s defences against bacteria (PRTN3, HP, SERPINB1, and RNASE3). In the remaining two cases (S4, S5), the responses associated with increased activity of the proteins related to defence processes prevail. In the case of S4 classified as CAL, which differed in structure from the rest, functional analysis showed a high content of calcium ion-binding proteins (MGP, MPO, S100A9), as well as statherin (STATH), the primary function of which is to stabilize saliva supersaturated solution and prevent the precipitation of calcium phosphate. The increased presence of proteins with activity related to the protection against microbes indicates that in the case of S4 and S5, microorganisms may be involved in the process of their formation, or the imbalance in the natural bacterial flora or a sudden bacterial infection could cause the formation of a deposit in the salivary glands. Based on preliminary quantitative analyses using the SWATH method, it was possible to perform a functional analysis of sialoliths leading to their grouping depending on the structure and the presence of antibacterial and inflammatory proteins. The group of proteins shared by all four calculi was visible, and the presence of proteins was characteristic for only one of them. For example, in S3, AZGP1, up-regulated, is described in the literature as stimulating lipid degradation in adipocytes and causing the extensive fat losses associated with some advanced cancers and antigen processing and presentation of endogenous peptide antigen via MHC class Ib [54]. The presence of, e.g., STATH in the qualitative and quantitative analysis of sialoliths is interesting. For the FASP method, we used a membrane with a mass cutoff of 10 kDa, which means that small proteins/peptides should be washed out of the preparation. It was also the case with most of the analysed samples. However, here the statherin was present and very well identifiable. The procedure repeated on other stone trials as well as by other persons gave the same results. STATH was present on the list of proteins identified in sialolith. Interestingly, the STATH Des [1,2,3,4,5,6,7,8,9,10,11,12,13] fragment was identified, and no spectra confirmed the presence of the N-terminus of the peptide responsible for calcium binding. Therefore, further research should check whether only a C-terminal fragment of statherin will be present in all the tested sialoliths. This fact also raises new questions: Is the presence of STATH in calculi due to the formation of complexes with another, larger protein, such as mucin, or is the proteolysis of statherin the cause of homeostasis disturbance in submandibular glands which starts the sialolith granulation process?

## 4. Materials and Methods

### 4.1. Protein Extraction

Five sialoliths removed during endoscopic, transoral, or transcervical procedures from submandibular glands were submitted for examination. One of them was intended for qualitative research (S1), the other four (S2–S5) for quantitative analysis. In the first step, each of the sialoliths was washed with 50 mM PBS saline buffer, dried, and broken up into smaller pieces with a scalpel and then ground in a porcelain mortar in liquid nitrogen. The resulting powder was poured into a clean Eppendorf vial. For the extraction process in the case of S1, two samples of 200 mg were prepared. For quantitative analysis, weights of 90 mg were prepared (S2–S5), and this value was determined by the smallest sialolith obtained for the tests (S5).

*Procedure 1.* The first batch of S1 was subjected to standard demineralization with 2.4 M hydrochloric acid. After the addition of acid, the sample was mixed by vortexing for 5 min and by ultrasonic bath at 37 °C for 30 min and left overnight in a refrigerator at 4 °C. After centrifugation for 25 min at 14,000× *g*, the solution was decanted (Fr I HCl), and the residue was treated with lysis solution (1% SDS, 100 mM Tris/HCl pH 8, 50 mM DTT), vortexed, and incubated at 95 °C for 10 min with vigorous shaking. After overnight incubation at 4 °C, the solution was vortexed for 10 min and kept in an ultrasonic bath for 10 min and then centrifuged in the same way as in the first case, decanted (Fr II Lysis). The residue was then treated a second time with lysis buffer, heated to 95 °C, and left overnight at 4 °C. After vortexing for 10 min and keeping the samples in an ultrasonic bath for 10 min, samples were centrifuged, and the supernatant solution was designated as fraction III (Fr III Lysis) and intended for qualitative research. To remove the hydrochloric acid, fraction I was transferred to a 3 kDa Amicon and washed five times with lysis buffer and then incubated at 95 °C for 10 min with vigorous shaking. Thus, all final fractions were in the same solution (the lysis buffer) before the next steps.

*Procedure 2.* For the second portion of S1 (200 mg), the order of treatment was reversed. First, the sample was treated twice with lysis buffer (stirring, heating 10 min 95 °C, 4 °C overnight, vortex, sonic bath, and centrifugation), which allowed Fr I and Fr II to be obtained. Finally, the residual precipitate was treated with 2.4 M hydrochloric acid and resulted in Fr III HCl after repeating the procedure. As in the first procedure, to remove the acid, dialysis was performed on 3 kDa Amicon in the lysis buffer, and the sample was heated for 10 min at 95 degrees before digestion.

Protein extraction for spectral library preparation and quantitative analysis for sialoliths S2–5 was performed according to procedure 1 (2.4 M HCl, lysis buffer x 2).

### 4.2. Protein Digestion and Final Clean-Up

The released proteins for all S1 fractions were digested on a 10 kDa membrane (Microcon, Millipore) according to the FASP procedure [55]. According to the procedure, the protein concentration in each fraction was measured spectrophotometrically before starting the digestion. An amount of 100 mg of protein for each fraction was taken for digestion. Generated tryptic peptides were desalted with homemade C18 StageTips according to the protocol described by Rappsilber [56]. For each desalting step, 10 µg of the peptides was taken and desalted on tip containing three layers of 3 M Empore C18 exchange disks. Peptides eluted by 100 µL of 60% acetonitrile/1% acetic acid/water were concentrated to 30 µL prior to MS analysis.

### 4.3. Mass Spectrometry Analysis

LC-MS/MS analysis was performed using a Triple TOF 5600+ mass spectrometer (SCIEX Framingham, MA, USA) coupled with the Ekspert MicroLC 200 Plus System (Eksigent, Redwood City, CA, USA). All chromatographic separations were performed on the ChromXP C18CL column (3 µm, 120 Å, 150 × 0.3 mm). The chromatographic gradient for each MS run was 7–35% B (solvent A 0% aqueous solution, 0.1% formic acid; solvent B 100% acetonitrile, 0.1% formic acid) in 30 min. The whole system was controlled by the SCIEX Analyst TF 1.7.1 software. Measurements for the spectral library were acquired in triplicate. Each cycle of the applied DDA method comprised precursor spectra accumulation in 100 ms in the range of 400–1200 *m*/*z* followed by top 20 precursor’s product ion spectra accumulation in 50 ms in the range of 100–1800 *m*/*z*, resulting in a total cycle time of 1.15 s. Formerly fragmented precursor ions were dynamically excluded.

### 4.4. Protein Identification

The MS and MSMS data obtained for S1 were analysed in PEAKS Studio software 10.0 (build 20190129). The basic settings included: instrument—TripleTOF, fragmentation method—CID, acquisition—DDA, precursor mass tolerance error 0.1 Da using monoisotopic mass, fragment ion tolerance 0.2, trypsin digestion, reduction, and alkylation of proteins (fixed modification—carbamidomethylation; variable modification—acetylation N-term, oxidation M), maximum allowed variable PTM per peptide 3. First, the data were searched against the *Homo sapiens* database (4 December 2020). Then, for the simultaneous identification of bacterial proteins, we used PEAKS Studio programs in the form of a Multi-Round Search engine for the additional database Bacteria (18 June 2020). Finally, the table of results included the human and bacterial proteins identified in the test sample.

### 4.5. Relative Quantitative SWATH MS Analysis

In order to perform the quantitative analysis, the sequential window acquisition of all theoretical mass spectra (SWATH MS) method was selected, allowing for a relative analysis of the protein content in the samples. Sialolith S2–S5 samples prepared according to protocol 1, digested in the FASP procedure (Microcon 3 kDa), and final clean up C18 were used to record the spectra in the SWATH MS mode. Experiments were performed in a looped product ion mode with the spectrometer set to high sensitivity focus. A set of 25 transmission windows of variable width was constructed using SwathTUNER software based on the equalized frequency of precursor ions and covering the precursor mass range of 400–1200 *m*/*z*. The collision energy for each window was calculated for +2 to +5 charged ions centred upon the window with a spread of five. The SWATH-MS survey scan was acquired in the range covered by constructed windows at the beginning of each cycle, with an accumulation time of 50 ms. Following SWATH-MS/MS spectra, product ion scans were collected in the range of 100 to 1800 *m*/*z* in 40 ms, which resulted in a total cycle time of 1.1 s.

### 4.6. Quantitative Data Analysis

For quantitative analysis of four sialoliths samples, a spectral library was created with the group file data processing in PeakView v. 2.2 (SCIEX), with settings described in detail by Lewandowska [57]. Joint search for library generation included all S2–S5 samples and pooled sample. For database search, ProteinPilot 4.5 software (Sciex) was used. It is based on the Paragon algorithm against the dedicated SwisProt *Homo sapiens* database (4 December 2020) with an automated false discovery rate. All files from SWATH experiments for sialoliths were downloaded to PeakView software and were processed with the previously established library. The resulting data were exported to an .xml file and exported to Marker View software. All data were normalized using the total area sums (TAS) approach. T-tests were conducted to find significant differences between protein concentration in tested samples. The mass spectrometry proteomics data were deposited to the ProteomeXchange Consortium via the PRIDE partner repository with the dataset identifier <to be deposited> [58]. Cytoscape 3.8.0 [59] and STRING 11.0 [60] were used for the interactome network visualization.

## 5. Conclusions

In conclusion, our preliminary studies show that the sialolith in addition to human proteins also contains a significant number of bacterial proteins. They come from natural bacteria found in the flora of salivary glands and pathogenic bacteria deposited with human proteins and inorganic material in the sialolith. Extending the work by including a microbiological database search in proteomic analysis will allow for the precise identification of pathogens and the determination of their role in protein deposition. The further refinement of the lysis buffer used in terms of increasing the lysis of bacterial cells may be a next step in research on the mechanism of sialolith formation, as well as being methodologically helpful in the extraction of proteins from any other solid material. We also showed that quantitative SWATH analysis is also useful for salivary stones analysis. The results of proteomic analyses correlate with the new classification of sialoliths, which in further studies may facilitate the determination of the mechanisms of their formation for individual patients. Our work is continuing, and we will soon present data for a larger group of patients.

## Figures and Tables

**Table 1:** Statistically significant sialolith proteins (*p*-value < 0.05) qualified based on quantitative analysis as candidates for biomarkers of the formation of salivary glands.

		Sialolith 2			Sialolith 3			Sialolith 4			Sialolith 5		
Protein ID	Group	*p*-Value	Fold Change	Log (Fold Change)	*p*-Value	Fold Change	Log (Fold Change)	*p*-Value	Fold Change	Log (Fold Change)	*p*-Value	Fold Change	Log (Fold Change)
P63261	Actin. cytoplasmic 2							0.0000556	7.284207094	0.862382285			
P59998	Actin-related protein 2/3 complex subunit 4							0.04393	8.260782456	0.917021185			
P02768	Albumin	0.03581	1.541531139	0.187952302									
P04745	Alpha-amylase 1	0.00116	0.418105336	−0.378714289	0.00073	0.212342882	−0.672962293	0.0000533	6.938583149	0.841270797	0.00174	0.276827038	−0.557791494
P01008	Antithrombin-III				0.03109	1.7503964	0.243136411				0.03258	2.069788471	0.315925964
P08311	Cathepsin G				0.03194	0.215401717	−0.66675084						
P28325	Cystatin-D							0.00014	12.34453279	1.091474658			
P01036	Cystatin-S							0.04307	3.873435826	0.588096365			
P01037	Cystatin-SN							0.04697	2.411624112	0.382309617			
P12724	Eosinophil cationic protein	0.02326	0.727721066	−0.138035053	0.0031	0.486055211	−0.313314396	0.0000154	4.412958749	0.644729868			
P11678	Eosinophil peroxidase	0.04349	0.419790017	−0.376967894									
P00738	Haptoglobin	0.02436	0.454821305	−0.3421592	0.00809	0.228983086	−0.640196596				0.00067	3.059357569	0.485630239
P69905	Hemoglobin subunit alpha	0.01515	0.30617954	−0.514023834				0.00391	0.015164453	−1.819173248	0.01928	1.689412748	0.227735767
P68871	Hemoglobin subunit beta	0.00904	0.542480283	−0.265616042				0.0000434	0.132372198	−0.878203219			
P02042	Hemoglobin subunit delta										0.02124	2.668392602	0.426249728
Q99880	Histone H2B type 1-L							0.04376	0.382441371	−0.417435133			
P62805	Histone H4	0.00074	0.615579981	−0.210715512	0.00077	0.569335782	−0.24463152	0.00023	3.057790931	0.485407788	0.03587	1.337430686	0.126271284
P02788	Lactotransferrin				0.03229	2.969429146	0.472672967				0.02921	0.215013951	−0.66753336
P30740	Leukocyte elastase inhibitor	0.00072	0.295559993	−0.529354352	0.00044	0.270358218	−0.568060425	0.0000901	8.758657111	0.942437525	0.0134	0.44069474	−0.355862133
P08493	Matrix Gla protein							0.00909	5.121921883	0.709432951			
P98088	Mucin-5AC	0.02361	0.35644789	−0.448003952	0.0198	0.357020126	−0.447307301				0.00386	3.117583194	0.493818052
Q8TAX7	Mucin-7	0.00921	1.683950908	0.226329426				0.01667	0.435705277	−0.36080718	0.0271	0.517439675	−0.286140275
P24158	Myeloblastin	0.04279	0.440744162	−0.355813431	0.012	0.300535469	−0.522104266	0.00643	4.525451976	0.65566196	0.02779	1.55929247	0.192927582
P05164	Myeloperoxidase							0.00425	6.591029535	0.818953258			
P80188	Neutrophil gelatinase-associated lipocalin							0.00821	8.288015407	0.91845055			
O75594	Peptidoglycan recognition protein 1							0.00758	0.184102407	−0.734940533	0.01342	1.71067952	0.233168656
P12273	Prolactin-inducible protein							0.04007	3.075452718	0.487909055			
Q6MZM9	Proline-rich protein 27							0.04956	5.728810271	0.758064439	0.01618	0.473683666	−0.324511591
P80511	Protein S100-A12							0.04541	0.426860545	−0.369713985	0.04279	1.814077348	0.2586558
P06702	Protein S100-A9							0.00073	2.087914099	0.319712627			
P02808	Statherin							0.02952	4.175986159	0.620759051			
P02814	Submaxillary gland androgen-regulated protein 3B	0.00181	2.030083123	0.307513821				0.00293	0.135815988	−0.867049102	0.0031	0.104953618	−0.979002587
P25311	Zinc-alpha-2-glycoprotein				0.03592	3.065769997	0.48653957						
Q96DA0	Zymogen granule protein 16 homolog B	0.02283	0.793156904	−0.100640891	0.04406	0.747544585	−0.1263629	0.00000717	3.008601253	0.478364632			

## Data Availability

The mass spectrometry proteomics data have been deposited to the ProteomeXchange Consortium via the PRIDE partner repository with the dataset identifier.

## Supplementary Materials

Table S1: List of proteins identified for S1 sialolith at FDR1 and FDR5, Table S2: SWATH-MS intensities after TAS normalization for all quantified proteins in individual and pool samples and the results of t-tests conducted for each individual sample compared with the pool sample.
